# Endogenous Calcification Inhibitors in the Prevention of Vascular Calcification: A Consensus Statement From the COST Action EuroSoftCalcNet

**DOI:** 10.3389/fcvm.2018.00196

**Published:** 2019-01-18

**Authors:** Magnus Bäck, Tamas Aranyi, M. Leonor Cancela, Miguel Carracedo, Natércia Conceição, Georges Leftheriotis, Vicky Macrae, Ludovic Martin, Yvonne Nitschke, Andreas Pasch, Daniela Quaglino, Frank Rutsch, Catherine Shanahan, Victor Sorribas, Flora Szeri, Pedro Valdivielso, Olivier Vanakker, Hervé Kempf

**Affiliations:** ^1^Translational Cardiology, Center for Molecular Medicine, Karolinska University Hospital Stockholmt, Stockholm, Sweden; ^2^Research Center for Natural Sciences, Institute of Enzymology, Hungarian Academy of Sciences, Budapest, Hungary; ^3^Department of Biomedical Sciences and Medicine, Algarve Biomedical Centre, Centre of Marine Sciences/CCMAR, University of Algarve, Faro, Portugal; ^4^LP2M, University of Nice-Sophia Antipolis and Vascular Physiology and Medicine, University Hospital of Nice, Nice, France; ^5^The Roslin Institute and Royal School of Veterinary Studies, University of Edinburgh, Edinburgh, United Kingdom; ^6^PXE Reference Center, Angers University Hospital, Angers, France; ^7^Department of General Pediatrics, Münster University Children's Hospital, Münster, Germany; ^8^Calciscon AG, Nidau, Switzerland; ^9^Department of Life Sciences, University of Modena and Reggio Emilia, Modena, Italy; ^10^British Heart Foundation Centre of Research Excellence, James Black Centre, School of Cardiovascular Medicine and Sciences, King's College London, London, United Kingdom; ^11^Laboratory of Molecular Toxicology, Veterinary Faculty, University of Zaragoza, Zaragoza, Spain; ^12^Department of Dermatology and Cutaneous Biology, Sidney Kimmel Medical College, Thomas Jefferson University, Philadelphia, PA, United States; ^13^Internal Medicine, Instituto de Investigación Biomédica (IBIMA), Virgen de la Victoria University Hospital, Universidad de Málaga, Málaga, Spain; ^14^Center for Medical Genetics, Ghent University Hospital, Ghent, Belgium; ^15^UMR 7365 CNRS-Université de Lorraine, IMoPA, Vandoeuvre-lès-Nancy, France

**Keywords:** arterial calcification, pyrophosphate, gla proteins, klotho, osteoprotegerin, osteopontin, fetuin

## Abstract

The physicochemical deposition of calcium-phosphate in the arterial wall is prevented by calcification inhibitors. Studies in cohorts of patients with rare genetic diseases have shed light on the consequences of loss-of-function mutations for different calcification inhibitors, and genetic targeting of these pathways in mice have generated a clearer picture on the mechanisms involved. For example, generalized arterial calcification of infancy (GACI) is caused by mutations in the enzyme ecto-nucleotide pyrophosphatase/phosphodiesterase-1 (eNPP1), preventing the hydrolysis of ATP into pyrophosphate (PP_i_). The importance of PP_i_ for inhibiting arterial calcification has been reinforced by the protective effects of PP_i_ in various mouse models displaying ectopic calcifications. Besides PP_i_, Matrix Gla Protein (MGP) has been shown to be another potent calcification inhibitor as Keutel patients carrying a mutation in the encoding gene or *Mgp*-deficient mice develop spontaneous calcification of the arterial media. Whereas PP_i_ and MGP represent locally produced calcification inhibitors, also systemic factors contribute to protection against arterial calcification. One such example is Fetuin-A, which is mainly produced in the liver and which forms calciprotein particles (CPPs), inhibiting growth of calcium-phosphate crystals in the blood and thereby preventing their soft tissue deposition. Other calcification inhibitors with potential importance for arterial calcification include osteoprotegerin, osteopontin, and klotho. The aim of the present review is to outline the latest insights into how different calcification inhibitors prevent arterial calcification both under physiological conditions and in the case of disturbed calcium-phosphate balance, and to provide a consensus statement on their potential therapeutic role for arterial calcification.

Vascular calcification (VC) is a common occurrence in patients affected with chronic diseases including diabetes, chronic kidney disease (CKD), or atherosclerosis. VC is also a hallmark of rare genetic diseases including pseudoxanthoma elasticum (PXE), generalized arterial calcification of infancy (GACI), Keutel syndrome, and progeria (1). Although the pathogenesis and clinical significance of VC are dependent on the etiology, the endpoint is invariably the formation of hydroxyapatite (HA) deposits in the arterial wall. Over the last two decades, studies have identified a number of calcification inhibitors in the healthy vessel wall that act to protect the vascular smooth muscle cells (VSMCs) from calcification. These factors act by either directly interfering with molecular pathways and/or sequestering hydroxyapatite components impairing their assembly and deposition. Their actions also depend on the stage of crystal formation and environmental context. Tremendous efforts have been put into the understanding of the mechanisms involved in the activity of these endogenous inhibitors that represent attractive factors with therapeutic relevance to VC treatment.

## Inorganic Pyrophosphate

Inorganic pyrophosphate (PP_i_), which consists of two inorganic phosphate molecules joined by a hydrolyzable ester, was first recognized as a key endogenous inhibitor of biomineralization in the 1960's (2). The major source of PP_i_ is extracellular ATP, which is released from cells through a highly regulated process (3). Subsequently, ATP can be rapidly hydrolyzed by ecto-nucleotide pyrophosphatase/phosphodiesterases (eNPPs) to produce PP_i_. Additionally, the membrane protein ANK (progressive ankylosis or ANKH) regulates PP_i_ levels through the transport of intracellular PP_i_ to the extracellular environment (4). Furthermore, a crucial source of systemic PP_i_ is provided through ATP-binding cassette subfamily C member 6 (ABCC6)-mediated ATP release from hepatocytes (5).

The formation of calcium phosphates and homogeneous precipitation is not thermodynamically favored in blood and solutions, but it still can take place through the nucleating activity of matrix proteins such as collagen or elastin (6, 7). Nucleation of amorphous calcium phosphate is prevented by PP_i_, which also inhibits the crystallization toward hydroxyapatite and crystal growth by binding to the hydroxyapatite surface (2, 8).

Reduced circulating PP_i_ concentration is commonly present during vascular calcification, as observed in hemodialysis patients (9). PP_i_ is hydrolyzed by local phosphatases, such as tissue-non-specific alkaline phosphatase (TNAP). Consequently, when the expression of TNAP is selectively increased, ectopic calcification is observed (10). During CKD, aortic calcification is accompanied by TNAP overexpression (11), an event that precedes the first observed calcium nanodeposits and hyperphosphatemia in a rat CKD model (12). In those, calcium deposition is followed by an unexpected local increase in ANKH expression and late increase in ENPP1 expression. These expression changes are followed by reduced plasma PP_i_ concentrations as a later event (12). Therefore, the local concentration of PP_i_ may be a relevant factor for the initial deposition of calcium in soft tissue, whereas reduced circulating PP_i_ levels may play a role during ESRD and hemodialysis.

A number of animal models have further contributed to our understanding of the role of reduced PP_i_ levels in VC. Mice lacking ABCC6 (*Abcc6*^−/−^) display a 40% reduction in plasma PP_i_ levels (13) and present arterial calcification and an enhanced myogenic response (14). *Enpp1*-knockout mice also show depressed levels of circulating PP_i_, with concomitant increased calcification in articular cartilage, peri-spinal ligament and aorta (15). A comparable phenotype can be found in the so-called tiptoe-walking (*ttw/ttw*) mouse (16), a naturally occurring mutant with a non-sense mutation in *Enpp1*, and the *asj/asj* mouse, which carries a V246D missense mutation (17). Furthermore, a naturally occurring truncation mutation of the C-terminal cytosolic domain of ANK appears to attenuate PP_i_ channeling in *ank/ank* mutant mice, which display VC (18). Intriguingly, intraperitoneal administration of PP_i_ in adenine-induced uremic calcification reduced calcium content by 70% (19), and a recent study has shown that orally administered PP_i_, also inhibits arterial calcification in *ttw/ttw* and *Abcc6*^−/−^ mice (20), reinforcing the central role of PP_i_ in the protection against VC.

## Gla Proteins

Matrix Gla protein (MGP) and Gla-rich protein (GRP), also known as Upper zone of growth plate and Cartilage Matrix Associated protein (UCMA) because of its original discovery in cartilage chondrocytes, are small secreted matrix proteins. They are members of the vitamin K-dependent (VKD) protein family containing, in their mature forms, several γ-carboxylated glutamate (Gla) residues. These VKD post-translational modifications (5 in human MGP, 15 in human GRP), enable MGP and GRP to bind calcium and calcified matrices (21, 22), which can modulate their function (23, 24).

Under normal physiological conditions, both MGP and GRP are synthetized by a variety of cell types including VSMCs and chondrocytes, where they function locally (21, 22). In agreement with this finding is the observation that both carboxylated and uncarboxylated MGP are localized at different levels in mineralized elastic fibers (25–27). Reverse genetics has clearly shown that MGP is a potent physiological inhibitor of calcification (28) since *Mgp*-deficient mice exhibit lethal early spontaneous medial calcification of their arterial trunk. Mutations in the human *MGP* gene cause Keutel syndrome, a rare autosomal recessive disease characterized by abnormal cartilage calcification, short stature, multiple peripheral pulmonary stenoses, brachytelephalangia, and inner ear deafness (29–31). However, in contrast to the mouse, humans rarely develop arterial calcifications (32). This has been suggested to be due to compensatory up-regulation of osteopontin (OPN, see below) in the vessel wall, which may have a protective effect in Keutel syndrome patients (33).

Interestingly, beside mutations, post-translational modifications (i.e., γ-carboxylation and/or phosphorylation for MGP) can further influence the clinical phenotype in patients. For MGP, its dephosphorylated and uncarboxylated form (dp-ucMGP) is a surrogate marker in CKD patients (34) and is associated with increased incidence of cardiovascular diseases (35, 36).

Several studies have also implicated GRP in vascular and soft tissue calcification, osteoarthritis, inflammation and carcinoma (37). Similar to MGP, GRP inhibits phosphate-induced VSMC calcification via SMAD-dependent BMP signaling (38). However, in contrast to *Mgp*-deficient mice, GRP deletion does not induce a clear phenotype (39), which contradicts a putative essential role as a physiological calcification inhibitor *in vivo*.

## Fetuin-A

Fetuin-A, also known as alpha2-Heremans-Schmid glycoprotein, is a liver-derived protein, which was initially isolated from fetal calf serum (40) and later also found in human serum (41, 42). Fetuin-A is the strongest circulating proteinaceous calcification inhibitor, being able to bind ~100 Ca^2+^ ions per molecule, i.e., ~50x the calcium-binding capacity of an albumin molecule (43). The Fetuin-A cystatin 1-domain contains a functional site, which is able to bind clusters of amorphous calcium phosphate (Ca_9_(PO_4_)_6_).

When pure Fetuin-A or Fetuin-A-containing serum is exposed to high calcium and phosphate concentrations, mineral-laden Fetuin-A molecules coalesce to form so-called primary calciprotein particles (CPP) (44, 45). These particles contain amorphous calcium phosphate and have a diameter of 50–100 nm. In analogy to lipoprotein particles, which solubilize fatty acids, CPP keep calcium phosphate in solution and prevent it from precipitating (46). Over time, however, primary CPP undergo spontaneous transformation toward secondary CPP, which are larger (>100 nm), of elongate shape and contain crystalline calcium phosphate (HA) (47). CPP can be regarded as the nano-morphological correlate of a *humoral mineral buffering system* in blood. Interestingly, both primary and secondary CPP have been found in blood samples from patients with CKD (48, 49). Recent work suggests that circulating CPP may predominantly represent primary CPP or even earlier forms (“low molecular weight CPP”) (50).

Consistent with the important calcification-inhibiting properties of Fetuin-A, mice deficient in *fetuin*-A develop heavy and diffuse soft tissue calcifications throughout the whole body (51). In contrast, upon induction of vascular injury, calcifications are primarily found in the intimal plaques, indicating an interaction between systemic and local calcification facilitators (51).

Fetuin-A is a negative acute phase protein, and, accordingly, its blood concentrations are commonly lower in the presence of inflammation (52). Furthermore, circulating Fetuin-A concentrations have been found to be associated with SNPs in the genetic region coding for the fetuin-A protein (53). Low fetuin-A concentrations have also been found in CKD patients and these low levels been associated with poor long-term cardiovascular outcome (54). Recent data indicate that fetuin-A should not be considered as an isolated factor only. In contrast, it should rather be seen in the functional context of the formation of mineral-fetuin-complexes/CPP and thus the performance of the humoral mineral buffering system (55, 56). Specifically, a newly developed blood test measures the transformation (T_50_-) time point from primary to secondary CPP *in vitro*, and thus the calcification homeostasis in blood beyond single factors. This provides more insight and functional information about the net effect of the humoral factors, which inhibit or promote calcification (57–61). These recent findings have the potential to vastly widen our view and to open new and exciting possibilities for research and clinical care alike.

## Klotho

Klotho is a single pass transmembrane protein that acts as a co-receptor for fibroblast growth factor-23 (FGF23) (62). Signaling through the Klotho and FGF receptor heterodimer decreases both phosphate reabsorption, via down-regulation of the renal proximal tubule type-II sodium phosphate co-transporters as well as 1,25(OH)2 vitamin D synthesis. Thus, Klotho plays a major role in calcium-phosphate equilibrium. Additionally, a soluble form of Klotho, produced by alternative splicing and cleavage by secretases, can be found in the circulation. This soluble form acts as an endocrine factor exerting its functions by its glycosidase activity (62) Soluble Klotho has been implicated in Wnt signaling inhibition (63) as well as maintenance of endothelial integrity (64).

Genetic deletion of *Klotho* in mice is characterized by a reduced lifespan, osteoporosis, arteriosclerosis, hyperphosphatemia, and ectopic calcification (65), hallmarks of CKD. Indeed, downregulation of Klotho is observed in CKD patients as well as in animal models of CKD (66–68). Interestingly, targeted deletion of *Klotho* in the murine kidney mimics the phenotype of the full body knockout mice (69). Taken together, these observations hence point to the kidney as the main producer and effector of Klotho in VC. However, transgenic overexpression of Klotho prevents CKD-induced medial calcification despite only modest serum phosphate reduction (67), suggesting that Klotho can also prevent medial calcification through alternative mechanisms other than reducing phosphate. Moreover, as mentioned previously, Klotho can act as an endocrine factor. This is further supported by the stable delivery of soluble Klotho to *Klotho*-deficient mice, which prevents VC despite a modest decrease in serum phosphate and an increase in serum calcium (70). In support of direct effects of Klotho in the vascular wall, treatment of rat VSMCs with recombinant soluble Klotho reduces both phosphate-induced calcification and sodium-dependent phosphate uptake (67). However, it is still debated if Klotho is endogenously produced by VSMCs (71). Therefore, whether these effects on VC are the consequence of circulating or locally produced Klotho remains unknown.

Two mutations in the α*KLOTHO* gene have been described in humans, which resemble the observed phenotype in mice. First, a homozygous missense mutation leading to an attenuated production of Klotho translated in hyperphosphatemia, hypercalcemia, and both vascular and ectopic calcification in the brain and the Achilles tendon (72). Second, a balanced chromosomal translocation in the proximity of the α*KLOTHO* gene resulted conversely in increased soluble Klotho levels, leading to hypophosphatemic rickets and skeletal abnormalities (73). In CKD, serum Klotho levels decrease alongside disease progression (74, 75). Moreover, in a small group of patients, urinary Klotho was decreased in stage 1 CKD patients, and the decrease correlated with the severity of the decline of the estimated glomerular filtration rate (67). However, in a prospective observational study of stage 2–4 CKD patients circulating Klotho levels did not predict atherosclerotic or acute heart failure events or death after 2.6 years of follow-up (76). It is worth noting that none of these studies explored the relationship between Klotho and VC. Nonetheless, decreased levels of circulating serum Klotho have been associated with increased arterial stiffness (77). In summary, serum and urinary Klotho could hence serve as predictors of CKD progression but not mortality, whereas their role as biomarkers for VC remains to be established.

## Osteopontin

Osteopontin (OPN) is a member of the SIBLING (**s**mall **i**ntegrin-**b**inding **li**gand, **N**-linked **g**lycoprotein) protein family of bone and teeth mineralization regulators (78). It is a multifunctional protein with a clear role in opsonization and chemotaxis via integrin signaling in non-mineralized tissues and is highly expressed by a variety of cell types including macrophages where it acts as a cytokines (79). Besides these roles, OPN was also one of the earliest regulators of mineralization identified in the vessel wall, although its mechanisms of action in regulating VC still remain incompletely resolved.

Independent studies identified OPN as a protein highly expressed in synthetic VSMCs in culture and subsequent studies *in vivo* identified OPN invariably at sites of mineralization in both atherosclerotic plaques and the vessel media (80–82). OPN. When expressed at sites of calcification, it forms a bridging protein that links the cellular extracellular matrix with mineral. It may also play a role in the dissolution of calcification by inducing macrophages to express carbonic anhydrase, which acts to acidify the local environment (83). Knockout mouse studies have shown that OPN is not an endogenous inhibitor of VC, as *Opn*-deficient mice do not develop spontaneous calcification, which is consistent with its low expression in contractile VSMCs (80, 82, 84). However, when *Opn*-deficient mice are crossed with *Mgp*-deficient mice or are subjected to a high phosphate diet, then calcification is exacerbated, suggesting that OPN functions as an inducible inhibitor of calcification (84, 85).

## Osteoprotegerin

Osteoprotegerin (OPG) is a protein endogenously expressed by contractile VSMCs. Its role in calcification was first identified when the *Opg-*knockout mouse was found to develop not only osteoporosis, but also VC and this was one of the first pathways linking these two age-associated pathologies (86). OPG acts as a neutralizing decoy receptor for RANKL and TRAIL and it has a major function in regulating osteoclast differentiation via this pathway (87). Mice lacking OPG develop osteoporosis because of increased osteoclast activity–however, the role of OPG in regulating VC has been more problematic to solve. Mouse OPG knockout studies showed that in the vessel wall the RANKL system is activated in the absence of OPG and this is associated with the presence of multinucleate osteoclast-like cells (88). *In vitro* studies have further elaborated the roles of OPG showing it can affect a number of cell types and processes including blocking osteoblastic change in VSMCs via direct and paracrine secretion from endothelial cells and this occurs via multiple signaling pathways (89, 90). OPG also appears to play an important role in the context of diabetes by regulating inflammatory responses (91, 92). Therefore, its actions in protecting the vessel wall from calcification may be context-dependent and clearly further work is required to delineate its multifunctional roles. Interestingly, epidemiological studies have shown that circulating levels of OPG are increased in patients with VC (93, 94). However, the significance of this biomarker remains unclear. It is not known whether its elevation reflects increased OPG to combat calcification, while the cellular origin of the circulating OPG has not been identified.

## Endogenous Vascular Calcification Inhibitor as Therapeutic Agents

The use and/or stimulation of the endogenous calcification inhibitors described herein constitute a tempting therapeutic strategy. Yet, limited data are available on successful attempts for the reversal of already established calcification.

The delicate balance of pro-calcifying P_i_ and the major anti-calcifying molecule PP_i_ (the P_i_/PP_i_ ratio) is regulated by numerous factors, opening up for intervention at several distinct levels. The dietary uptake of P_i_ can be hindered by phosphate binders (e.g., sevelamer and aluminum salts) or novel therapies (e.g., tenapanor), which inhibit P_i_ absorption from the gastrointestinal (GI) tract leading to a decreased P_i_/PP_i_ ratio. These molecules are used in hyperphosphatemia in patients suffering from CKD (95, 96).

Besides decreasing P_i_, the P_i_/PP_i_ ratio could potentially be reduced through elevating blood PP_i_ levels. This can be experimentally achieved via the intraperitoneal or oral administration of PP_i_ in rodent models, the latter being effective in humans as well (20, 97, 98). Although oral delivery has clinical potential as it halts crystal growth in the PXE or progeria mouse models (97, 98) and prevents calcification even as a gestational treatment in the GACI mouse model (20), it might have several limitations. First, only ~0.1% of dietary PP_i_ is absorbed (20) as presumably the vast majority of PP_i_ is degraded in the GI tract by the microbiome. Second, dietary PP_i_ and P_i_ intake are variable particularly as PP_i_ is a frequently used food additive (E450). Additionally, considering the short half-life of PP_i_ in plasma, several daily doses of PP_i_ might be necessary, although repetitive administration of PP_i_ might lead to GI and other side effects (19, 20). Therefore, maintaining sufficient P_i_/PP_i_ plasma levels might be difficult to obtain via oral administration. However, analogs of PP_i_, the bisphosphonates, are already in clinical use for the treatment of osteoporosis, despite the rare but severe adverse effects (e.g., jaw necrosis). Moreover, bisphosphonates have been shown to reduce ectopic calcification in patients with GACI (99, 100) or PXE (101), and in animal models of PXE and CKD (17, 97, 102).

Besides direct administration of PP_i_ or uncleavable derivatives, alternative strategies could target endogenous enzymes involved in the maintenance of PP_i_ concentration. The serum PP_i_ level can thus be increased by the recombinant soluble Enpp1 enzyme, as shown in laboratory conditions (103). Finally, a novel promising target is TNAP, which cleaves PP_i_ into two P_i_ ions increasing thereby the P_i_/PP_i_ ratio and the propensity for calcification (104, 105). SBI-425 is a recently developed specific TNAP inhibitor (106), with sufficient oral bioavailability and efficacy in mouse models (103–105, 107).

## Consensus Statements

Endogenous calcification inhibitors represent a crucial defense mechanism against VC. Although the function of the endogenous VC inhibitors has been extensively studied, there are still some important clues lacking to fully elucidate their role in the development of VC. To attain this knowledge, the EuroSoftCalcNet COST Action consortium here emphasizes the following:

1. The deep phenotyping of genetic alterations in calcification inhibitor pathways in both humans and mice represents a powerful tool to better define their clinical and therapeutic relevance and to increase our understanding of the alteration of the pro- and anti-calcifying balance during different stages of VC and the influence of local and systemic inhibitors on the cellular response of VSMCs and/or the physical-chemical properties of mineral deposit.
2. The calcification inhibitors need to be studied from an integrated point of view, including detailed analysis of the molecular pathways and the interactions involved, the relation to altered phosphate (P_i_/PP_i_) balance and the association with different calcification phenotypes. Altogether, this would help identify an ideal biomarker measure that should reflect calcification homeostasis beyond single factors.
3. The central role of the P_i_/PP_i_ ratio in the regulation of VC makes PP_i_ an interesting candidate as an effective and low-cost treatment against VC.
4. The exploration of the therapeutic potential of PP_i_ and other calcification inhibitors should focus on bioavailability and tolerability as well as efforts to avoid bone loss as a consequence of stimulating these pathways within a long-term treatment perspective.

## Author Contributions

All authors listed significantly participated in the content and writing of the article. MB and HK contributed to the conception of the article, and to the writing, reviewing, and editing of the manuscript.

### Conflict of Interest Statement

AP is an inventor of the calcification propensity (T50) test, and an employee and stockholder in Calciscon AG (Nidau, Switzerland) which commercializes this blood test. The remaining authors declare that the research was conducted in the absence of any commercial or financial relationships that could be construed as a potential conflict of interest. The reviewer WJ declared a past co-authorship with one of the authors CS to the handling editor.
